# Molecularly Imprinted Polymers: Shaping the Future of Early-Stage Bone Loss Detection—A Review

**DOI:** 10.1021/acsomega.3c08977

**Published:** 2024-02-15

**Authors:** Bala Agnishwaran, Geetha Manivasagam, Anjaneyulu Udduttula

**Affiliations:** † Centre for Biomaterials, Cellular and Molecular Theranostics (CBCMT), Vellore Institute of Technology (VIT), Vellore-632014, Tamil Nadu, India; ‡ School of Bio Sciences and Technology (SBST), Vellore Institute of Technology (VIT), Vellore-632014, Tamil Nadu, India

## Abstract

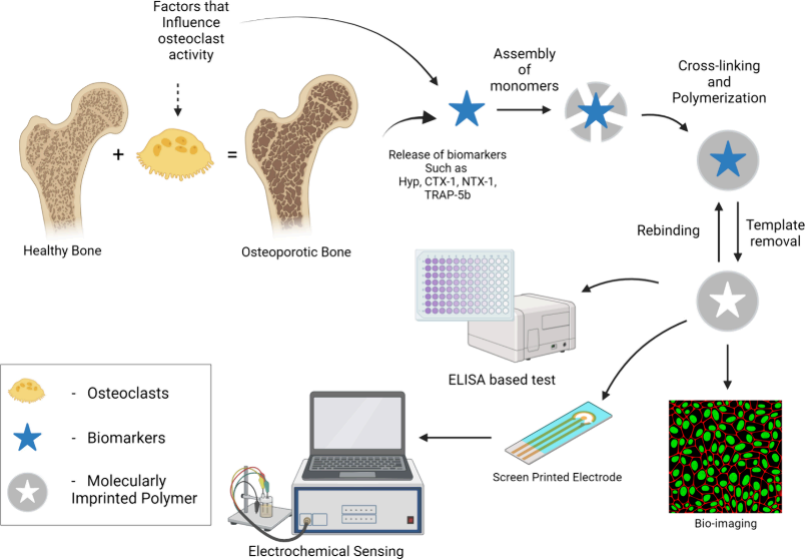

Osteoporosis is the deterioration of bone mineral density (BMD) because of an imbalance between bone resorption and formation, which might happen due to lots of factors like age, hormonal imbalance, and several others. While this occurrence is prevalent in both genders, it is more common in women, especially postmenopausal women. It is an asymptomatic disease that is underlying until the first incidence of a fracture. The bone is weakened, making it more susceptible to fracture. Even a low trauma can result in a fracture, making osteoporosis an even more alarming disease. These fractures can sometimes be fatal or can make the patient bedridden. Osteoporosis is an understudied disease, and there are certain limitations in diagnosing and early-stage detection of this condition. The standard method of dual X-ray absorptiometry can be used to some extent and can be detected in standard radiographs after the deterioration of a significant amount of bone mass. Clinically assessing osteoporosis using biomarkers can still be challenging, as clinical tests can be expensive and cannot be accessed by most of the general population. In addition, manufacturing antibodies specific to these biomarkers can be a challenging, time-consuming, and expensive method. As an alternative to these antibodies, molecularly imprinted polymers (MIPs) can be used in the detection of these biomarkers. This Review provides a comprehensive exploration of bone formation, resorption, and remodeling processes, linking them to the pathophysiology of osteoporosis. It details biomarker-based detection and diagnosis methods, with a focus on MIPs for sensing CTX-1, NTX-1, and other biomarkers. The discussion compares traditional clinical practices with MIP-based sensors, revealing comparable sensitivity with identified limitations. Additionally, the Review contrasts antibody-functionalized sensors with MIPs. Finally, our Review concludes by highlighting the potential of MIPs in future early-stage osteoporosis detection.

## 1 Introduction

Bone is a living tissue that undergoes constant remodeling by continuous resorption and reformation modulated by osteoclast and osteoblast cells. An imbalance in this process where the osteoclast activity is higher than the osteoblast activity may result in bone loss. Inactivation of osteoblast cells might also lead to bone loss. Bone loss is normal in people after the peak bone mass is obtained around the third decade. Women will lose around 35% of their cortical bone and 50% of their trabecular bone.^1,2^ The bone constituents are measured by measuring the bone mineral density, which is expressed in units of T score, where a T score of −1 or higher is considered normal, a T score ranging from −1 to −2.5 is considered osteopenia, and a score below −2.5 is considered osteoporosis.^3^ Bone loss disease includes deteriorating conditions like osteopenia, osteoporosis, and osteoarthritis, which is the degeneration of cartilage.^4^ This Review discusses bone structure, formation, resorption, remodeling, and bone loss, with an emphasis on bone loss, its biomarkers, and the detection of bone loss biomarkers using molecularly imprinted polymers (MIPs) for early-stage osteoporosis.

### 1.1 Structure and Significance of Bone

**Figure 1:**
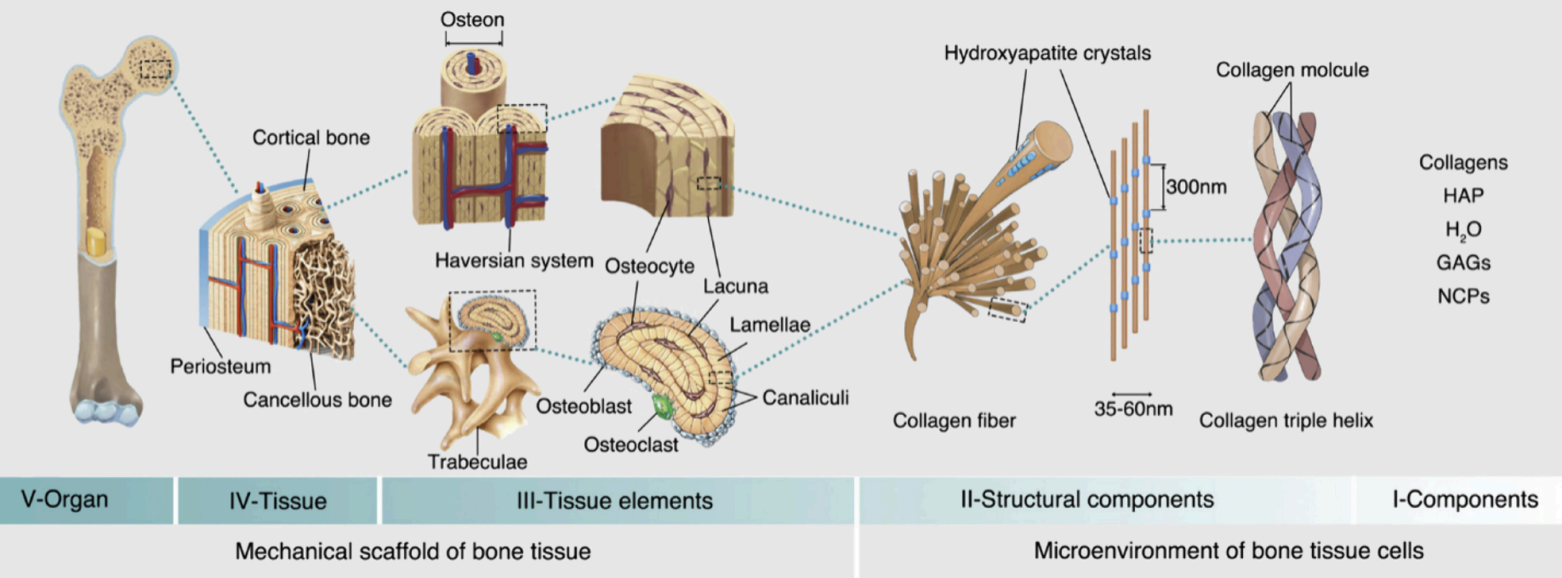
Structure of bone from its whole structure down to its microarchitecture (from left to right), breaking down its structure sequentially as an organ, tissue, tissue elements, microstructures, and its basic components. Reproduced from ref (13) and reprinted from the Creative Commons Attribution 4.0 license (CC BY-NC-ND 4.0 DEED).

Bone is a complex metabolically active biological tissue composed of both organic and inorganic substances, comprising cells, an organic extracellular matrix with well-structured collagen fibrils, nanocrystalline structures, and inorganic rodlike minerals of sizes ranging from 25 to 50 nm.^5,6^ These collagen fibrils orient themselves into sheets called lamellar sheets. These lamellar sheets are further organized into osteons, which are structures that contain blood vessels surrounded by concentric layers of lamellar sheets,^6^ and these osteons are densely packed to form the cortical bone as shown in Figure 1. Bone tissues are of two types: cortical bone and cancellous bone. Cortical bone is made up of 20% water, 45% bone salts, and 35% organic substances, most of which are collagen type 1.^7^ Cancellous bones are spongy bones that are called trabecular bones; they are light and porous and enclose a large space that gives a honeycomb appearance. Cancellous bones make up 20% of the human skeleton, providing structural support and flexibility, and they are more metabolically active than cortical bones as they inhabit more of the cells and cellular constituents.^8^ The cortical bone has an inner endosteal surface surrounded by a fibrous tissue called the periosteum, and the joints are lined by articular cartilage.^9^ Bones provide structural support to the body, protect vital organs, and provide an environment for bone marrow.^9^ They are active tissues that undergo constant remodeling to adapt to various conditions to provide mechanical support to the body, and they are constantly resorbed by osteoclasts and rebuilt by osteoblasts, respectively. Any imbalance in the resorption and formation ratio (more resorption than formation) might result in bone loss.^10^ They also act as a mineral reservoir to store ions that are required by the body, mainly calcium and phosphate, which can be released into the bloodstream when needed.^11^ The mineral density increases steadily as the formation is faster than resorption and reaches its maximum density at the age of 30–35, called peak bone mass, beyond which it starts diminishing.^10^

Ossification is a process by which bones are formed in two main stages: primary and secondary ossification.^12^ Based on its structure, bone is divided into many types, namely long, short, flat, irregular, and sesamoid-shaped bones; these bones are formed in various mechanisms during embryonic development.^11^ Long bones like the femur, ulna, tibia, and radius, as their name suggests, are long and have two terminals called epiphysis and a hollow cavity in the middle called diaphysis. They both originate from different, independent ossification centers separated by a layer called growth plates.^11^ Short bones, carpal and tarsal bones, found in hands and feet are cube -shaped bones with a spongy bone interior surrounded by a single layer of cortical bone.^11^ Flat bones include the skull, sternum, and scapula, which contain spongy bones sandwiched between compact bones. Irregular bones are vertebral bones, and ethmoids have a thin outer cortical layer surrounding the inner spongy bone. Sesamoid bones are short bones that are embedded inside the tendons.^11^

## 2 Cellular Mechanisms of the Bone

Bone tissue is composed of osteoblasts, which are cells responsible for bone formation; osteocytes which reside in the bones; and a bone matrix that contains hydroxyapatite, collagen, and noncollagenous proteins like proteoglycans, sialoproteins, and 2HS-glycoproteins. These components together form the complex tissue called bone.^10^

Mesenchymal cells from bone marrow give rise to osteoblasts via the Wnt/j3-catenin signaling pathway.^10^ These mononucleated cells are responsible for the formation and mineralization of bone matrix, and they are responsible for the maintenance of skeletal architecture. As the bone forms, these cells secrete osteoid, which eventually mineralizes into bones. As a result, some of the osteoblast gets trapped in the bones and turns into osteocytes. The osteocytes communicate with one another using extensions of their plasma membrane, acting as mechanosensors and instructing osteoblasts and osteoclasts when to form or absorb the bone.^10,15^ Osteoclasts secrete sclerostin, which is a glycoprotein that can inhibit osteoblast activity by binding to LRP5/6 and interfering with the Wnt pathway, thus acting as a negative regulator.^16^

### 2.1 Bone Formation and Its Signaling

As mentioned before, bones are formed by a process called ossification. There are two processes of ossification: intramembranous ossification and enchondromal ossification.^10^ The formation of the bone happens in a few steps. The steps involved in intramembranous ossification are the formation of the ossification center, followed by calcification, the formation of trabeculae, and the development of the periosteum, whereas in enchondromal ossification, the steps involved in formation are the development of the cartilage, the growth of cartilage, the development of the primary ossification center, followed by the development of the secondary ossification center, and finally the formation of the articular cartilage and epiphyseal plate.^10^ Bones are formed by osteoblast cells that originate from mesenchymal stem cells; these mesenchymal stem cells give rise to osteoprogenitors, which then differentiate into osteoblasts. Osteoblasts produce Type 1 collagen and proteoglycans, along with some noncollagenous proteins like osteonectin, osteopontin, osteocalcin, and bone sialoprotein. They secrete cytokines and colony stimulating factors like interleukin-6 (IL6), interleukin-11 (IL11), granulocyte-macrophage colony stimulating factor (GM-CSF), and macrophage colony stimulating factor (M-CSF). Growth factors like bone morphogenetic proteins (BMPs), insulin-like growth factors (IGFs), transforming growth factor-beta (TGFβ), and platelet-derived growth factors (PDGFs) are secreted by osteoblasts; mature osteoblasts have parathyroid hormone (PTH) and 1,25-dihydroxyvitamin D receptors, which play a major role in the regulation of bone metabolism and mineral homeostasis.^11^

**Figure 2:**
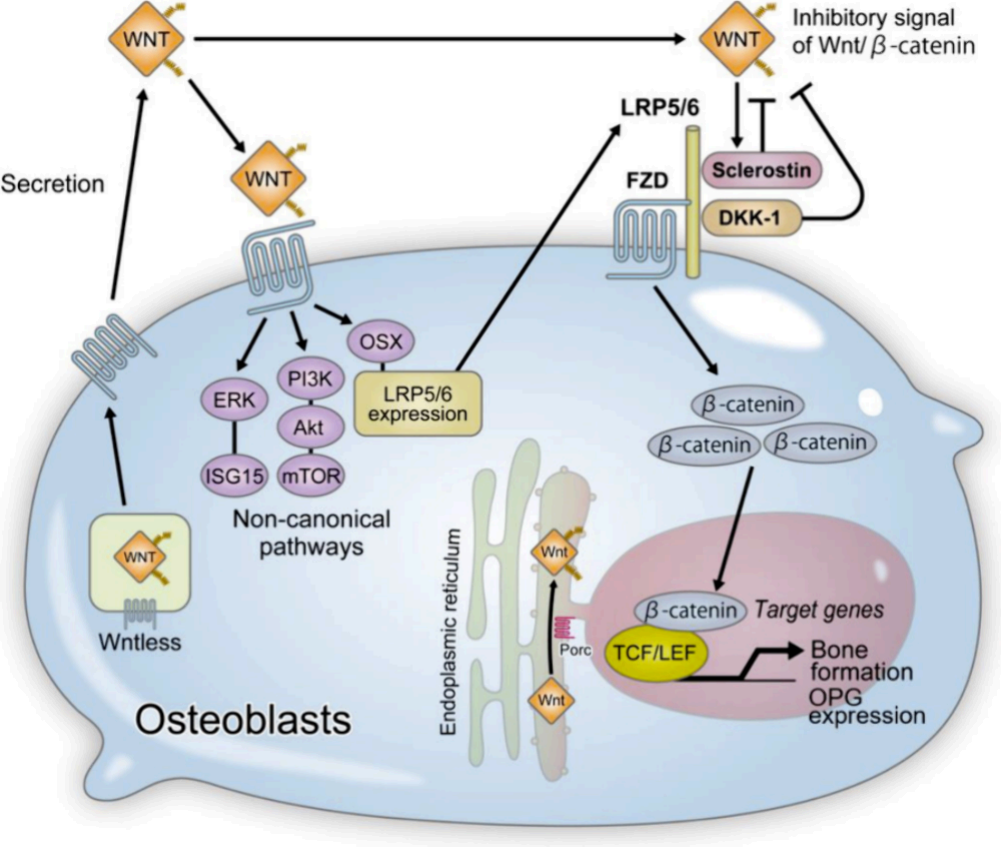
Wnt activates β-catenin-dependent canonical and β-catenin-independent noncanonical pathways; the β-catenin-dependent signal promotes bone formation by inducing osteoblastogenesis and OPG expression, while the β-catenin-independent pathway enhances the expression of LRP5/6, promoting osteoblast differentiation. Reproduced from ref (16) and reprinted from the Creative Commons Attribution 4.0 license (CC BY).

Wnts are a family of extracellular glycoproteins that activate different intercellular signaling pathways; they are responsible for cell fate determination, proliferation, polarity, migration, and gene expression,^15,17^ and they play a major role in osteoblastogenesis. The Wnts set in motion three distinct cascades of intracellular signaling pathways: the Wnt/Ca^2+^ pathway, the Wnt/β-catenin pathway, and the Wnt/planar polarity pathway. Cell fate determination, proliferation, and survival are promoted by the canonical pathway (Wnt/β-catenin pathway) by altering gene expression through transcription factor lymphoid enhancer factor/T cell factor (Lef/Tcf) and an increase in β-catenin levels. The pathway is activated by the binding of Wnt to the transmembrane receptor frizzled (Fz) and low-density lipoprotein receptor-related protein 5/6 (LRP5/6) coreceptors; the absence of the Wnt ligand suppresseses the activation of Wnt responsive genes^15^ as shown in Figure 2. Inappropriate activation of Wnt signaling might result in cancer, while reduced levels of Wnt signaling might result in osteoporosis. BMPs are bone morphogenic proteins that are growth factors that provide essential signals for full osteoblastogenic differentiation. Several genes are expressed in the osteoblasts that are responsible for signaling, maintaining the matrix, and mineralization; they are osteocalcin, which is responsible for signaling and controlling the differentiation of osteoclasts; and osteopontin and bone specific alkaline phosphatase (ALP), which are stabilizers of the matrix and other regulatory proteins.^15^ In addition to the pathway, signaling pathways like hedgehog signaling, notch signaling, and PI3K-Akt might also contribute to bone formation.^11,18,19^ Runx2 plays an important role in determining the fate of osteoblast cells, confirms mesenchymal cells toward osteoblast lineage, and helps in the expression of several genes in osteoblast cells.^14^

**Figure 3:**
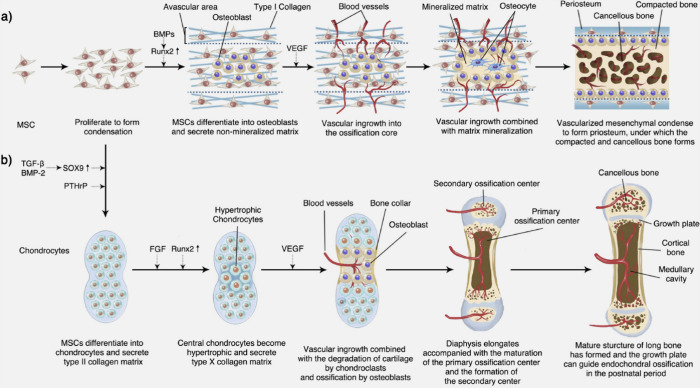
Bone formation, depicting intramembranous ossification (a) and enchondromal ossification (b). Reproduced from ref (13) and reprinted from the Creative Commons Attribution 4.0 license (CC BY-NC-ND 4.0 DEED).

Bone formation is initiated when osteoblasts are differentiated from osteoprogenitors, which organize around a point called the ossification center. These mesenchymal cells proliferate and form a condensed mass around a capillary network and differentiate into osteoblast cells, which form the osteoid. The osteoid eventually mineralizes and traps the osteoblasts, which are then converted into osteocytes. They are encircled by active osteoblasts which continue the process. The trabeculae gradually thicken, making the intervening spaces form the cancellous bone. This formation continues where the surrounding mesenchymal cells condense to form the periosteum along its edges and surfaces. A network of woven bone is initially formed, which is then replaced with lamellar bone. This is the process of intramembranous ossification.^10^ The process of intramembranous ossification and enchondromal ossification is shown in Figure 3.

Enchondromal ossification occurs in the formation of long bones and natural fracture healing mechanisms. They begin with points in cartilage called primary ossification centers, which occur during fetal development. Development of short bones, diaphysis of long bones, and certain parts of irregular bones occur by enchondromal ossification. The epiphysis of long bones and the terminals of irregular bones and flat bones develop after birth by secondary ossification. In early fetal life, all the long bones seen are a form of hyaline cartilage, which resembles the bone shape. These cartilages are surrounded by a periosteum, and these cartilages continue to grow and produce more extracellular matrix with the development of chondroblasts. Ossification occurs at the primary center of ossification; the perichondrium becomes the periosteum, which contains the osteoprogenitors. The osteoblasts secrete osteoid, the chondrocytes at the center of the primary ossification stop secreting collagen and other proteoglycans and start secreting alkaline phosphatase, the calcification occurs, and the hypertonic chondrocytes induce the sprouting of blood vessels by secreting vascular endothelial cell growth factor, which branches out and starts transporting cells needed for the formation. New osteoblast cells use the calcified matrix as a scaffold and secrete osteoid to form the trabecula. The osteoclasts break down the spongy bones to form the medullary cavity.^10^

### 2.2 Bone Resorption and Its Signaling

Unlike osteoblast cells that originate from mesenchymal stem cells, osteoclasts originate from myeloid cells of the hematopoietic lineage; they are multinucleated cells formed by the fusion of mononuclear cells of the immune system. Osteoclastogenesis is the process of the formation of osteoclasts, and it is initiated by two main molecules: macrophage colony stimulating factor (M-CSF) and receptors for activation of nuclear factor kappa B and its ligands (NF-kB, RANK, and RANKL).^20^ RANKL is a membrane -bound protein of osteoblasts, bone marrow stromal cells, smooth muscle cells, and activated T lymphocytes. They belong to the family of tumor necrosis factor (TNF) cytokines, and the expression of RANKL in osteoblasts contributes to the activation, differentiation, and maturation of osteoclast cells.^15^ Macrophage colony stimulating factor (M-CSF) stimulates macrophages to proliferate and become osteoclast progenitors, and RANKL stimulates these cells to differentiate into functional osteoclasts. Osteoblast cells produce osteoprotegerin (OPG), which is a member of the TNF-α receptor family that acts as a decoy receptor that prevents RANK and RANKL interaction by binding to it and inhibiting its activity, thus regulating osteoclastogenesis.^11^

The bone resorption begins with the attachment of osteoclasts to the target site; it is modulated by integrin receptors with the help of vitronectin to form a ruffled membrane, which is a complex infolding of the plasma membrane that only appears when attached to a bone.^20^ Once attached, it forms a microenvironment around the target site and starts its acid secretion, which is its central activity. Vacuolar (V-type) electrogenic H^+^-ATPase is highly expressed, which transports protons through the plasma membrane to the sealed area, creating an acidic environment. This acidic environment dissolves the hydroxyapatite into the environment, exposing the organic matrix.^21^ The digestion of collagen and other organic substances is done by Cathepsin K, which is a proteinase synthesized by the osteoclast cells. The degraded materials are uptaken by the osteoclasts, packed into vesicles, and released on the other side of the cells into the stream.^21,22^ Bone resorption is modulated by various pathways and happens due to multiple factors like calcium homeostasis, wear and tear, damage repair, and much more. Irrelevant activation of osteoclasts might lead to bone loss.^21^ Estrogen is known to reduce osteoclastic activity; it promotes the expression of OPG, which acts as a decoy receptor for RANK, inhibiting the activity of osteoclast cells.^23^ Lack of estrogen can also result in bone loss.

### 2.3 Bone Remodeling Pathway

**Figure 4:**
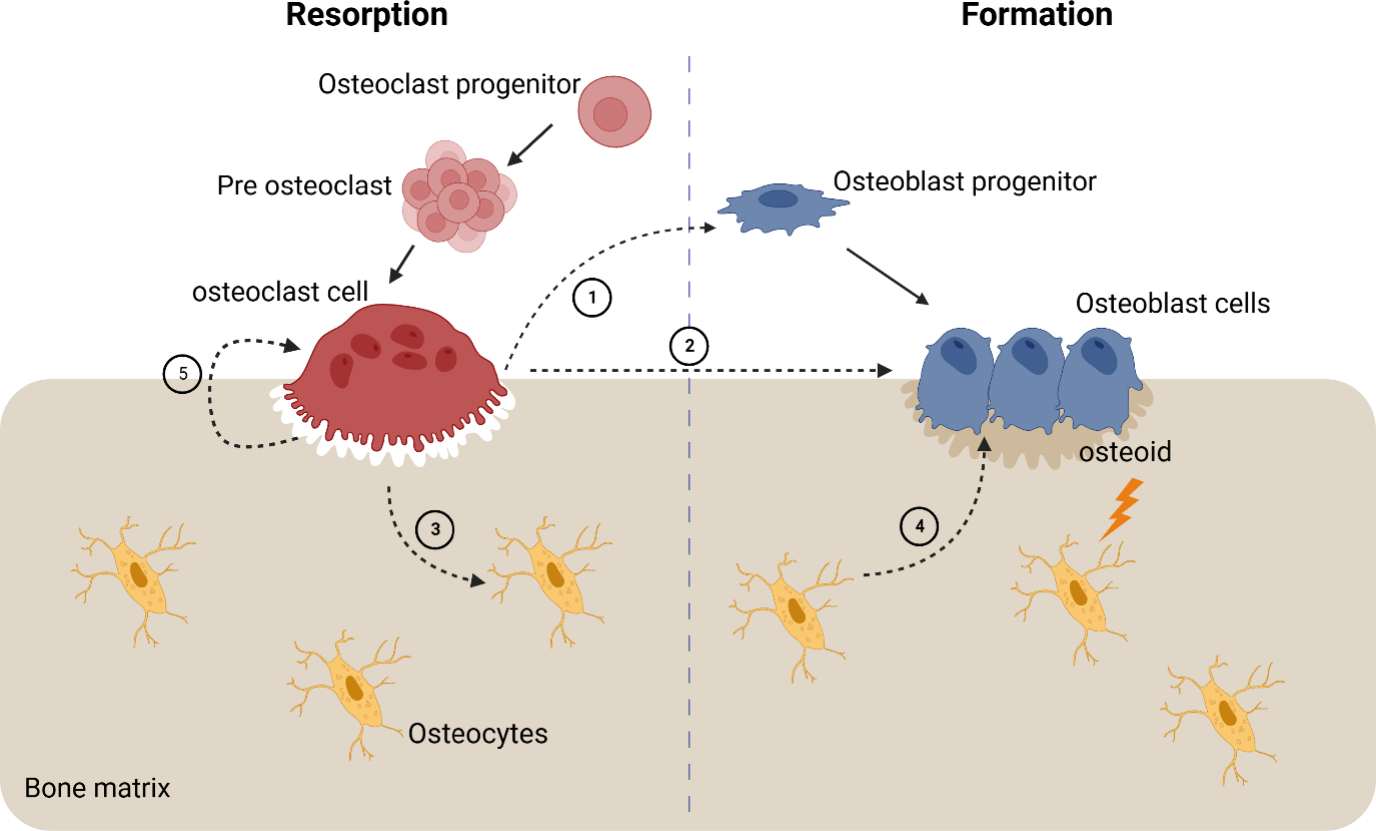
Coupling mechanism of the bone starting with osteoclast cells signaling cells of osteoblast lineage cells in the canopy, osteoblast progenitors, (1) reversal cells in the bone surface, and osteoblast cells (2); it also signals the osteocytes in the bone matrix (3) which signal the osteoblast cells (4); physical changes in the bone are also signaled to the osteoblast cells to secrete and form the appropriate amount of bone matrix. Adapted from ref (28).

Remodeling is a process that involves both osteoblast and osteoclast cells. It occurs in small packets of cells called basic multicellular units (BMUs). These BMUs occur at different and separate sites from one another, and at any given time, 20% of the cancellous bone surface is being remodelled.^24^ The number of active sites of bone formation and resorption determines the rate of bone turnover. Remodeling is done to improve structural support; mechanical stress can alter this bone architecture. Recent studies have shown that mechanical stress can be sensed by osteocytes, and they secrete paracrine factors like interleukin-like growth factor-1 (IGF 1) in response.^21^ In a remodeling cycle, IGF 1 might initiate the reaction, but the process is called a coupling mechanism as shown in Figure 4. The reaction starts with resorption, followed by a reversal phase and bone formation by osteoblast cells.^24^ The coupling mechanism ensures that the bones resorbed and bones formed are equal.^26^ There are some hormones that might affect remodeling: parathyroid hormone, insulin, growth hormone, calcitonin, 1, 25-dihydroxyvitamin D3, and growth factors like IGF 1 and 2. Transforming growth factors like fibroblast growth factors (FGF) and bone morphogenic proteins (BMPs) might also influence bone remodeling.^24^ The osteoclasts initially resorb the bone, as in the resorption stages discussed earlier, and the osteoclast cells undergo apoptosis induced by TFG-β.^24^ This is then followed by the reversal phase, where the osteoclast detaches from the site and osteoblast cells attach to the site to form the new bone. This event is coordinated by various regulators.^24^ Any abnormalities in this balance between osteoblasts and osteoclasts and this coupling reaction might lead to bone disorders like osteopetrosis, osteoporosis, and much more.^27^

## 3 Bone Loss Disease

### 3.1 Osteoporosis: Characteristics and Epidemiology and Risk Factors

Osteoporosis is a bone loss disease that affects the bone mineral density of individuals, rendering them brittle and susceptible to fracture.^29^ It is a complex systemic disease that involves various pathways that can deteriorate the bone mineral density (BMD), affecting the microarchitecture of the bone.^30^ Osteoporosis occurs in both genders, but women are more prone to osteoporosis, especially postmenopausal women, due to the reduced levels of estrogen. There are also other factors that contribute to the disease, including age, low BMI, family history, geography, and lifestyle choices.^30^ According to the WHO (World Health Organization), 30% of postmenopausal women suffer from osteoporosis. Studies reported that in India, 61 million individuals are affected by osteoporosis, of which 80% are women.^30^ Osteoporosis is an asymptomatic disease that remains undetected until the occurrence of a fracture. Osteoporotic bones only need minimal or lesser trauma to break, and these fractures can lead to hospitalization. The individual may be bedridden for life, and these fractures can sometimes be fatal.^27^ Osteoporosis is characterized by the deterioration of bones, making them porous. It is referred to as the silent killer as it is asymptomatic, and the condition is undetected until the incidence of a fracture.^31^ The bone mineral density (BMD) reaches its maximum at around 30 years of age and will slowly start to deteriorate.^27^ The bone can become weak and porous, increasing the risk of low-trauma fractures. Even coughing or bending too much can induce a fracture.^32^ It is classified into primary osteoporosis and secondary osteoporosis. Factors like age and hormonal imbalances like estrogen and testosterone can result in primary osteoporosis, while secondary osteoporosis is triggered by calcium imbalance, vitamin D, and inflammatory reactions.^27^ Primary osteoporosis is further classified into two types: Type 1 and Type 2.^29^ Type 1 is known as postmenopausal osteoporosis, which involves a population of women above the age of 65 and affects around 5–25% of early menopausal women.^25^ Smoking, glucocorticoid treatment, rheumatoid arthritis, prolonged immobility, organ transplantation, type 1 diabetes, hyperthyroidism, chronic liver disease, and obstructive pulmonary disease are some risk factors for osteoporosis.^29^

### 3.2 Diagnosis and Management of Osteoporosis

Bone density is measured by measuring the bone mineral density using dual X-ray absorptiometry to predict fracture risk^33^ where the T scores of the individuals are evaluated. Diagnosis begins with checking patient history for risk factors associated with osteoporosis. A physical examination along with a complete history of the patient is carried out, and physical findings such as nodular thyroid, hepatic enlargement, jaundice, and cushingoid might reveal the secondary factors for osteoporosis.^34^ After the assessment, the T scores of the BMD are measured with dual-energy X-ray absorptiometry (DXA), which is the gold standard for osteoporosis diagnosis. They are expressed in g/cm.^2,29,34^ The test compares and detects bone strength, which is directly correlated to fracture risk. DXA measurements done in the hip and lumbar regions and the spine are the major predictors of low BMD.^29^ Prediction of fractures in recent days has been done using algorithms such as the fracture risk assessment tool model (FRAX), which is a computational model designed to predict the probability of a fracture. It requires the complete details of the patient, including a history of the disease and the drug interventions provided to assess the risk.^35^ Clinical assessment of osteoporosis is done using enzyme-linked assays like ELISA, which can be used to detect low levels of biomarkers found in the blood and urine. Radioimmunoassay is like ELISA but done with radiolabeled antibodies. As analyzing bone mineral density can take a long time to study bone turnover, biomarkers can be an easier way of detecting any changes in bone turnover in a quick and efficient manner.^36^ Clinical assessment of osteoporosis along with BMD measurement can give reliable and detailed information about resorption, formation, and bone turnover, which can help assess the condition better.^37^ Biosensors are implied in sensing, detecting, and monitoring bone loss. Yun et al. developed an enzyme free impedance sensor for monitoring bone resorption using CTX-1.^38^ Liu et al. developed a point of care device for the detection of ALP activity to access and monitor bone turnover,^39^ and studies have also been done for other sensors to detect bone activity.^40^

## 4 Bone Disease Biomarkers

**Figure 5:**
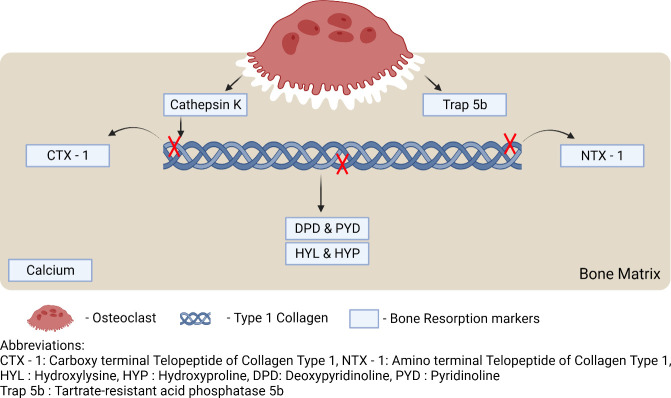
Biomarkers of bone resorption. Adapted from ref (55).

Bone turnover can also be assessed by analyzing specific biochemical markers found in the blood and urine. These biomarkers are produced because of bone formation and resorption and released into the bloodstream. These biomarkers are indicators of bone formation and resorption.^41^ Biomarkers are indicators of a biological process that not only can help to understand the normal process but can also be used to analyze pathogenic processes, responses to drugs, and other abnormal processes.^35^ These clinical biomarkers can be used in the detection, monitoring, and evaluation of osteoporosis using biochemical tests, which can help in the assessment of the risk and management of osteoporosis.^42^ Various bone turnover biomarkers are shown in Figure 5 including bone formation, resorption, and regulatory markers.

### 4.1 Bone Formation Biomarkers

These biomarkers are byproducts of bone formation. Total alkaline phosphatase (ALP), osteocalcin, bone specific alkaline phosphatase (BALP), procollagen type 1 C-terminal propeptide (P1CP), and procollagen type 1 N-terminal propeptide (P1NP) are some biomarkers^37^ that can indicate bone formation. ALP levels in the blood can indicate the presence of liver, bone, and gall bladder disorders.^37^ ALP is a prominent product of osteoblasts and their precursors.^42^ Osteocalcin is a noncollagenous protein that plays a major role in calcium homeostasis, metabolic regulation, and mineralization. Secreted by hypertonic chondrocytes and mature osteoblasts, it is regulated by 1,25-dihydroxtvitamn D_3_.^42^ Osteocalcin is a significant biomarker for bone formation.^37^ P1NP and P1CP are released into the bloodstream as a result of the conversion process of collagen from procollagen by the action of proteases.^37^

### 4.2 Bone Resorption Markers Detection

Similarly, when bone is being resorbed, some biomarkers are released, which can reveal the activity of bone resorption. The detection of carboxy-terminal cross-linked telopeptides of collagen type 1 (CTX-1), hydroxyproline (HYP), tartrate-resistant acid phosphatase 5b (TRAP 5b), pyridinoline, deoxypyridinoline (DPD), Cathepsin K, and amino-acid-terminal cross-linked telopeptides of type 1 collagen (NTX-1) can indicate the activity of bone resorption.^43^

#### 4.2.1 Hydroxyproline

Hydroxyproline is a result of post-translational hydroxylated proline; hydroxyproline makes up 12–14% of collagen and is released into the stream as a result of collagen degradation during bone resorption. Almost 90% of it is metabolized by the liver, but an elevated amount of hydroxyproline is found in urine samples of postmenopausal women.^44^ Ten percent of hydroxyproline is derived from newly synthesized procollagens, and it can also be found in collagen found in other tissues, so hydroxyproline is a nonspecific biomarker for osteoporosis.^37^ Urinary hydroxyproline can be measured using HPLC, according to Simsek et al.^45^ Jagtap et al. studied the urinary hydroxyproline levels of postmenopausal women using the Bergman and Loxley method and indicated that postmenopausal nonosteoporosis women have less urinary hydroxyproline than postmenopausal osteoporotic women.^46^

#### 4.2.2 Hydroxylysine

Galactosyl hydroxylysine and glucosyl-galactosyl-hydroxylysine are the two forms of hydroxylysine. Hydroxylysine is a derivative of post-translationally hydroxylated lysine, and galactosyl hydroxylysine is more bone specific. They are released during collagen degradation, and the presence of galactosyl hydroxylysine in urine samples indicates bone resorption.^47^ Cascio studied bone fragility in postmenopausal women using several biomarkers, with a high emphasis on galactosyl hydroxylysine, using the HPLC technique and concluded that it can be a significant biomarker for osteoporosis.^47^

#### 4.2.3 Deoxypyridinoline and Pyridinoline

Deoxypyridinoline (DPD) is a bone specific biomarker, as it is present in dentin and bone.^48^ Pyridinoline and deoxypyridinoline cross-link collagen peptides, stabilizing the collagen structure, and are released into the bloodstream when collagen is degraded during resorption. Pyrodinoline is also a cross-linker but is found in cartilage, bones, blood vessels, and ligaments and is a nonspecific biomarker when compared to DPD.^37^ Bettica et al., in their study, compared the detection of pyridinoline and deoxypyridinoline using HPLC, discussed its limitations, and concluded that the method is time-consuming and tedious.^49^

#### 4.2.4 Bone Sialoprotein

Bone sialoprotein is a noncollagenous protein and an essential part of the extracellular matrix of the bone, comprising about 8% of noncollagenous proteins. It is generated by osteoblasts and is an integral part of the cell matrix adhesion process; it also stimulates osteoclast-mediated bone resorption.^37^ Fassbender et al. studied the correlation between bone sialoprotein and other significant biomarkers. They determined the levels of bone sialoprotein using radioimmunoassay and concluded that it is a significant biomarker for bone resorption.^50^

#### 4.2.5 Tartrate-Resistant Acid Phosphatase 5b

Osteoclast cells secrete TRAP 5b during bone resorption, which is released into the bloodstream, metabolized in the liver, and excreted in urine. The presence of TRAP 5b in blood and urine can indicate the activity of osteoclasts; it is a specific biomarker and is studied widely due to its specificity, and TRAP 5b levels can be evaluated using immunoassays.^37^ Shidara et al. studied biomarkers for osteoporosis along with TRAP 5b using fragment-absorbed immunocapture enzymatic assay (FAICEA) to validate it as a bone resorption biomarker.^51^

#### 4.2.6 CTX-1 and NTX-1

Carboxy-terminal (C-terminal) cross-linked telopeptides of type 1 collagen and amino-terminal (N-terminal) cross-linked telopeptides of type 1 collagen are released into the bloodstream during bone resorption because of osteoclastic activity. Elevated levels of CTX-1 and NTX-1 can indicate increased bone resorption. The protease released by the osteoclasts cleaves collagen at specific sites to release CTX-1 and NTX-1 into the bloodstream. NTX-1 can stay stable in urine for 24 h and can be detected using ELISA-based tests. As food intake can influence the presence of CTX-1 in the bloodstream, NTX-1 is preferred as a biomarker.^37^ ELISA tests can be used to determine the levels of CTX-1 in the serum. A monoclonal antibody can be developed against the octapeptide sequence found in the α-1 chain in the β-isoform.^37^ A study by Baim and Miller showed that CTX-1 has high specificity for the detection of osteoporosis, and CTX-1 levels in serum can indicate bone resorption activity.^52^

#### 4.2.7 Interleukin 6

Interleukin 6 (IL6) is an inflammatory cytokine. IL6 influences osteoclastogenesis, and increased levels of IL6 might result in excess bone resorption. Thus, detecting IL6 in the body can indicate the resorption activity.^53^ Hormones like estrogen control the action of IL6, but in the case of postmenopausal women, due to the lack of estrogen, the IL6 activity might increase, leading to the differentiation of cells toward the osteoclast lineage and thus increasing bone resorption, leading to bone loss.^26^ IL6 also suppresseses the production of slerostin in osteocytes, which indirectly influences bone turnover.^12^ Jabber et al. evaluated salivary IL6 as a potential biomarker using an ELISA test and studied postmenopausal women and their IL6 levels, concluding that IL6 can be a potential biomarker for osteoporosis.^54^

## 5 Limitations on Current Diagnostic Techniques

The current method of osteoporosis diagnosis and fracture risk assessment uses multiple methods discussed before. Measuring bone mineral density for the detection of osteoporosis by DXA is the current gold standard, but it gives us two-dimensional images of three-dimensional structures, which may not be a proper representation of bone strength.^33^ Osteoporosis is only evident in radiographs after 30% of bone minerals have deteriorated.^56^ Algorithm-based FRAX tools require a detailed history of the patient and sometimes family information too, which may not be available all the time.^35^ Imaging like CT and MRI instrumentation requires large infrastructure, which may not be available everywhere.^33^ Clinical assessment for osteoporosis can help us identify the presence of the biomarkers mentioned above with the help of assays like ELISA and colorimetric analysis,^37^ but early-stage detection of these biomarkers still poses a challenge. Biomarkers like CTX-1, NTX-1, and IL6 can be analyzed using ELISA^37^ methods. Studies have been done to estimate and validate salivary IL6 as a potential biomarker,^54^ but the kits and synthesis of monoclonal antibodies for the specific biomarkers can be very expensive,^36^ and most of the rural population could not afford such things. Standard methods for diagnosis and early-stage detection, cost, and reliability of tests remain a challenge in studying osteoporosis. Recent studies have been focusing on miRNA-based detection for osteoporosis, but they are still in the research state.^57,58^ Studies have also been done to understand the relation between bone loss and serum homocysteine levels.^59^Table 1 presents a comprehensive overview of the constraints associated with existing diagnostic methods as well as the limitations inherent in certain techniques that are currently undergoing research and development.

**Table 1:** Limitations on Current and Ongoing Research on Techniques for Early Bone Loss Diagnostics

sample no.	technique	detection stage	limitation	reference
1	dual X-ray absorptiometry	early stage	limited sensitivity, minimal availability of instruments, and radiation exposure	(29)
2	quantitative ultrasound (QUS)	early stage	unreliable results; still needs secondary confirmatory tests	(60)
3	infrared thermography	early stage	requirement of specialized equipment	(61)
4	microwave imaging	early stage	still in development	(62)
5	clinical biomarker analysis	early stage	expensive and requires expertise	(37)

## 6 Molecularly Imprinted Polymers (MIPs)

**Figure 6:**
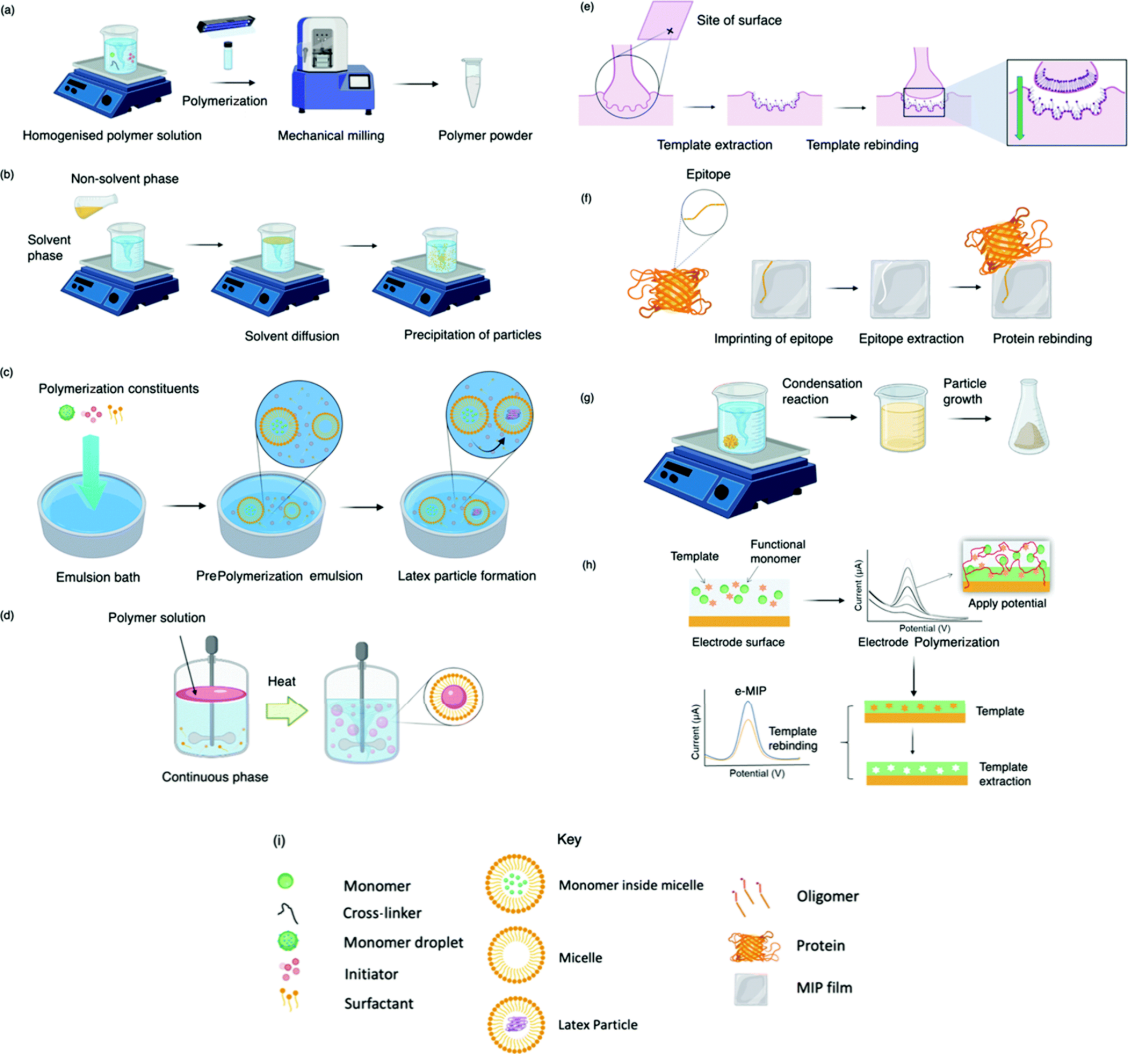
Molecularly imprinted polymers synthesized using various methods like bulk synthesis (a), precipitation synthesis (b), emulsion (c), suspension (d), surface printing (e), epitope printing (f), sol–gel method (g), and electropolymerization (h). (i) Key for each technique. Reproduced from ref (66) and reprinted from the Creative Commons Attribution 3.0 license (CC BY 3.0).

Molecularly imprinted polymers (MIPs) are artificial antibodies that can mimic and function like antibodies, but they are more robust and specific in nature than natural antibodies. They are polymers synthesized using molecular imprinting technology that have high specificity, selectivity, and sensitivity toward the target molecule. They are developed as analogues for natural antigen–antibody interactions that can be used for various applications like sensing, separating, and drug delivery.^63,64^ They are polymerized along with the target analyte as a template; the template is then removed, leaving the polymer with a cavity specific to that analyte.^62^ They are more stable than natural or synthesized antibodies, which require specific transport and storage conditions, whereas MIPS can be stored at any temperature. The cost of synthesis of MIPs is also low when compared to the cost of antibodies.^64^ They mimic the functions of natural antibodies and biological receptors but are more robust synthetic materials.^63^ MIPS can compensate for the current challenges faced when manufacturing antibodies, transporting them, and addressing stability issues.^66^ In the first step, the templates interact with the monomers through hydrogen bonding, reversible covalent bonding, van der Waals force, and electrostatic interaction with the functional groups.^67^ The monomers interact and bind with the template in the presence of a cross-linker. After polymerization, the template is removed, leaving the polymer with a cavity specific to its shape, size, and chemical functionality.^63^ MIP synthesis begins with the choice of an appropriate monomer for the template based on its interaction, which is crucial for the polymerization process and recognition of this molecule. The assembly of monomers around the template, forming a cavity that is secured by cross-linking agents, forms a three-dimensional polymeric network,^66^ and the interaction between the template and the polymer network forms the molecular recognition site. The selectivity of the MIPs is validated by comparing the binding of MIPs with NIPs (nonimprinted polymers) of the same composition where nonspecific binding to the MIP surface is evaluated.^66^ The synthesis process is low-cost and easy, and the resulting polymers are more versatile and stable and can resist degradation in a wide range of pH, solvents, and temperature, which allow them to be used in synthesis with biological templates like proteins, amino acids, nucleic acids, peptides, and drugs.^67^ The usual method of synthesis involves using a template, a functional monomer, a cross-linker, and an initiator, and the reaction is run on a porogenic solvent.^63^ There are different methods of polymerizing MIPs, which include bulk synthesis, phase inversion, soft lithography, suspension, emulsion, precipitation, sol–gel method, epitope imprinting, electropolymerization, and several other methods as described in Figure 6.^64,66,68^

### 6.1 MIPs in Sensing and Clinical Diagnosis

**Figure 7:**
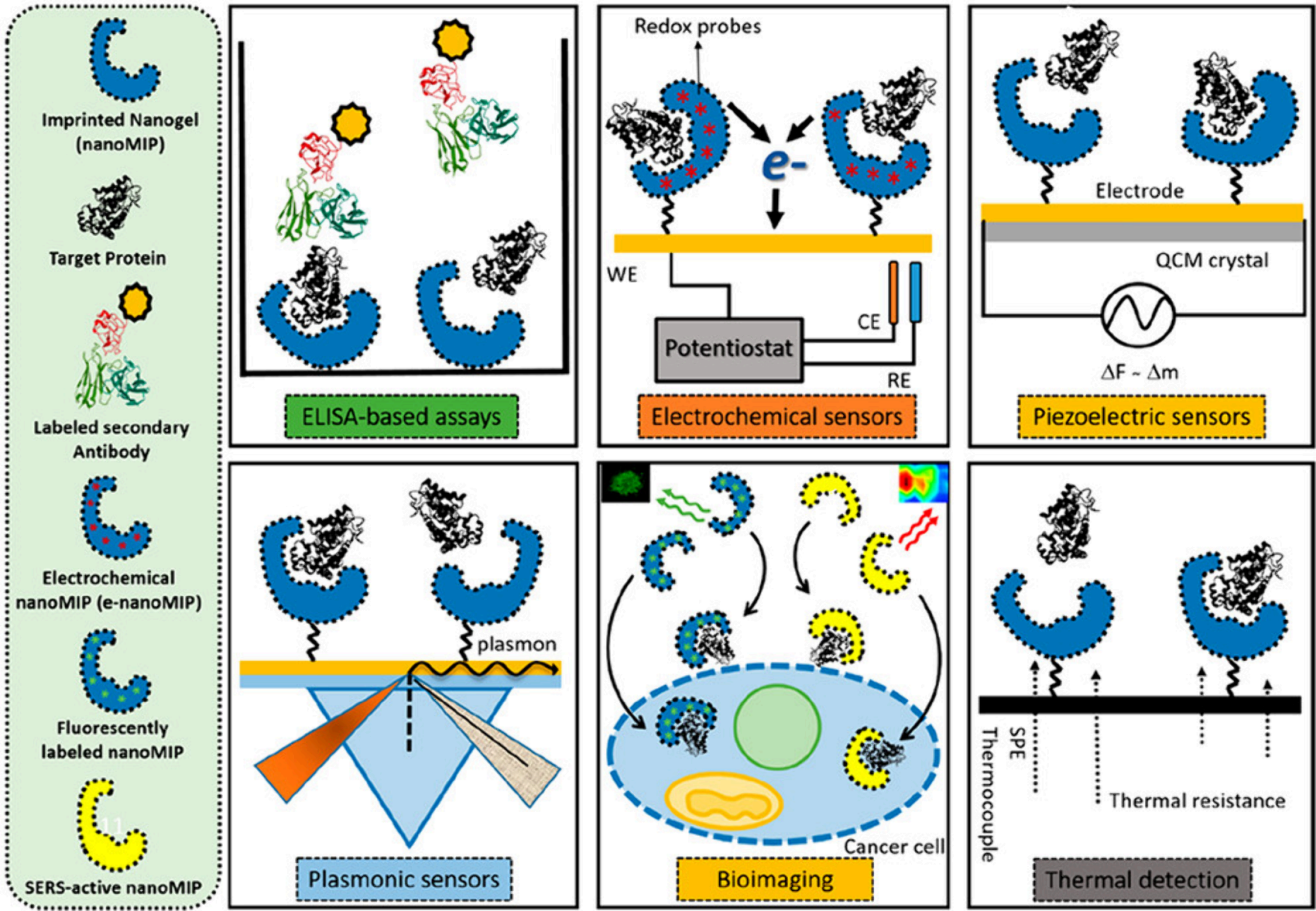
Various methods by which MIPs can be incorporated into sensing for the identification of analytes in the sample. Reproduced from ref (65) and reprinted from the Creative Commons Attribution 4.0 license (CC-BY 4.0).

As the roles of MIPs are based on their binding potential, they can be used in various sensing applications like chemicals, food, metals, clinical biomarkers, and microbial contaminants. Due to its specificity, it can be used in the clinical diagnosis of various biomarkers; they can be integrated into the sensing surface of a biosensor using electrodeposition, polymerizing, or grafting methods to functionalize the sensor to work as an electrochemical sensor. MIPS can be integrated into various electrodes and quartz crystal microbalances (QCM) to detect the analyte based on piezoelectric differences in the sensing surfaces.^63^ The molecular imprinted sorbent assay is a pseudoimmunoassay that is like ELISA but replaces the antibody with synthetic MIPs, and it is comparatively as efficient as the actual assay.^63^ MIPS can be integrated with fluorescent, colorimetric, surface plasma resonance, and surface-enhanced ramen scattering (SERS) optical sensors for the detection of analyte molecules. They can be coated on screen-printed electrodes to analyze the test sample in a cost-effective manner.^68^ MIPs incorporated with sensors can help us detect various bodily functions, normal vs elevated levels of biomolecules, pathogenic infections like bacterial and viral infections, drug responses, drug dosages, inflammatory responses, etc.^66^ MIPs incorporated with quantum dots (QDs) can be used in bioimaging applications where the surface epitope can be used as a template to synthesize MIPs incorporated with QDs. These nano-MIPS can be administered to target that epitope and, once bound, can be imaged.^68^ Cecchini et al. developed nano-MIPs incorporated with quantum dots to monitor the Human Vascular Endothelial Growth Factor (hVEGF), which is overexpressed in cancer cells, and have proven that nano-MIPs can specifically target the secreted factor, which can be used in imaging cancer cells.^69^ Similarly, MIPs can be imprinted with bone loss biomarkers incorporated with quantum dots to help monitor bone activity. Battaglia et al. demonstrated that MIPs can be used in pseudo-ELISA immune assays by replacing the capture antigen with imprinted procalcitonin on a (polynorepinephrine)-based polymer and proved that it can be a viable technique.^70^ Various ways in which MIPs can be incorporated into sensing are represented in Figure 7.

#### 6.1.1 MIPs-Based Biosensors

MIPs incorporated into various sensing surfaces and signal transducers can help estimate the presence of biomarkers and biochemicals in biological fluids like blood, serum, urine, and saliva. Detection of biomarkers using sensors can be a cost-effective way of detecting, estimating, and monitoring body functions when compared to standard clinical tests, which can sometimes be an expensive and time-consuming process and sometimes require experts in handling samples. Biosensors can be operated with minimal training and can be accessible in wider areas as compared to standard techniques. Also, MIPs incorporated into sensors can be produced at a lower cost when compared with other biosensors that are functionalized by antibodies and other conjugates. Diouf et al. developed a technique to detect urinary creatinine, elevated levels of which can indicate nephritis and renal dysfunction. They imprinted creatinine on a gold screen-printed electrode using an acrylamide-based polymer and tested it using differential pulse voltammetry and electrochemical impendence spectroscopy. This MIP-based sensor has detected the presence of the target analyte in the urine sample.^71^ Lopes et al. developed MIPs based on naltrexone and noroxymorphine to monitor naloxone levels in biological samples. They electropolymerized the monomers on the surface of a screen-printed carbon electrode and used the differential pulse voltammetry method to detect the target molecules in the samples. The study showed that the developed sensor can be used in biological samples.^72^ Koyun et al. developed a surface plasmon resonance-based sensor for the detection of human-activated protein C. They imprinted DNA aptamer and hAPC and coated the gold surface with polymers. The sensor is characterized by contact angle analysis, atomic force microscopy, and ellipsometry analysis. They concluded that real-time analysis of biological samples can be achieved by using this sensor.^73^ Zhou et al. developed a fluorescence-based sensor by incorporating MIPs with graphene quantum dots to give a fluorescent readout. They developed this sensor to detect dopamine in serum and urine samples. The fluorescent readout was measured by spectroscopic methods, and they concluded that this can be used as a cost-effective method for detecting targets in clinical samples.^74^ Kartal et al. incorporated MIPs into a quartz crystal microbalance to develop a piezoelectric sensor for the selective determination of insulin. They reported that the MIP-QCM-based sensor is a viable tool for diagnosing and analyzing clinical samples.^75^ The above-discussed sensors and their capabilities are represented in Table 2.

**Table 2:** Strategies Employing MIPs for bBiomarker Detection^a^

method of detection	mode of detection	target	sensitivity–limit of detection (LOD)	reference
pseudo-ELISA	MIPs as capture antigen	equine and canine PCT	5.87 and 4.46 ng/mL	(70)
electrochemical sensor	gold screen-printed electrode by DPV and EIS	creatinine	0.016 and 0.81 ng/mL	(71)
	carbon screen-printed electrode	naltrexone and noroxymorphine	∼75 and 192 ng/mL (0.20 and 0.67 μM)	(72)
optical sensor	surface plasmon resonance	DNA aptamer and hAPC	1.5 and 5.2 ng/mL	(73)
fluorescent sensors	graphene quantum dots-based fluorescent detection	dopamine	∼0.38 ng/mL (2.5 nM)	(74)
piezoelectric	quartz crystal microbalance	insulin	0.00158 ng/mL	(75)

a Abbreviation: hAPC: human-activated protein C, DPV: differential pulse voltammetry, EIS: electrochemical impendence spectroscopy, PCT: procalcitonin.

### 6.2 MIPs-Based Detection Strategies for Bone Loss

Biomarkers released during bone resorption like CTX-1, NTX-1, TRAP 5b, and hydroxyproline and inflammatory biomarkers like IL6 can be imprinted using molecular imprinting technology and can be used as a detection method for osteoporosis. Afsarimanesh et al. developed an antibody-based sensor for CTX-1 detection and compared the values with standard ELISA-based tests. The results show that the developed sensor’s sensitivity and detection levels are almost on par with standard ELISA-based tests, with the detection levels of standard tests of samples being 0.6229 and 0.5280 ppb for the sensor-based test and 0.6514 and 0.5049 ppb for the standard ELISA tests, with an error value of 4.3% and 4.6%, respectively.^76^ The same researchers developed an MIP-based sensor for the detection of CTX-1 in biological samples; they functionalized an interdigital sensing surface using MIPs imprinted with CTX-1 and characterized the sensor using the SEM and EIS methods. Serum samples were analyzed by using the developed sensor and compared with the standard ELISA test, with the detection limits being 1.416, 0.184, 0.98, and 0.453 ng/mL for the developed sensor and 1.465, 0.187, 0.100, and 0.450 ng/mL for the standard ELISA test, with an error value of 3.3, 1.6, 2.0, and 0.6%, respectively. The limit of detection of the developed MIP-based sensor is found to be 0.09 ng/mL, and the ELISA test is 0.02 ng/mL, which indicates that the standard ELISA method is superior to the built sensor in terms of sensitivity and detection, but the MIP-based sensor is a reliable and cost-effective method for the detection of CTX-1 in biological samples.^77,78^ Similarly, another study done by Jesadabundit et al. used l-hydroxyproline as a template for the synthesis of MIPs with two monomers and electropolymerized it on a screen-printed electrode surface for bone loss detection. The rebinding of the template was estimated using a cyclic voltammetry and electrochemical impedance spectroscopy. The results were compared with HPLC analysis on spiked serum samples. The sensor’s standard deviation was around 2.14–4.62%, with the limit of detection being 0.13 μg/mL.^79^ Though MIPs-based sensing can seem like a reliable technique, it is still inferior to antibody-based sensing as it requires lots of optimization and standardization in synthesizing and sensing,^63^ and most of the functional applications of MIPS are still in the research and development stage.

## 7 Conclusion and Future Prospects

Bone is an intricate living tissue that is continuously being reconstructed and remodelled to adapt to varying mechanical loads and stress experienced by it. The formation, resorption, and remodeling of the bone are done by osteoblasts and osteoclast cells, which originate from mesenchymal and hematopoietic lineages. The differentiation, commitment, activation, maturation, and deactivation are modulated by various pathways, inflammatory cytokines, signaling molecules, and several other factors like stress. An imbalance or disturbance in any of these reactions might cause the bone to overdevelop or underdevelop which can cause various diseases like osteopetrosis, osteopenia, and osteoporosis. Osteoporosis is an underlying, nondetectable disease that can be characterized by the depletion of bone mineral density and the deterioration of bone mass. The occurrence of osteoporosis makes the bone weak, brittle, and more vulnerable to fracture. Even regular activities like bending too much, sneezing, coughing, and low trauma can cause fractures, which are severe cases of osteoporosis. Since osteoporosis is an understudied disease that affects many people, especially women, there is an increasing need for early-stage detection of osteoporosis, as detecting it at an early stage can help prevent possible and occurring fractures and provide a proper intervention and prevention strategy for individuals diagnosed with osteoporosis even before the fracture occurs. Currently, existing detection methods for osteoporosis have limitations, cost concerns, and availability problems. Techniques like DXA have infrastructural limitations, while clinical techniques require trained personnel to carry out techniques like HPLC and ELISA. These techniques can also be expensive, and running many samples continuously can be a painful and troublesome process. MIPs can provide an alternative solution by making the detection method easy, cost-effective, reliable, portable, and available even in areas without high infrastructure and hospital and transportation facilities. MIP-based sensors can be operated with little to no training, they can be manufactured in bulk, and they can be transported easily to health centers where clinical samples like serum, urine, and saliva from patients with a risk of osteoporosis can be analyzed for the presence of bone loss biomarkers. MIPs can be synthesized by imprinting clinical biomarkers of osteoporosis. These MIPs can be incorporated into various sensors, as discussed above, like screen-printed electrodes, surface plasmon resonance sensors, QCM-based piezoelectric sensors, and fluorescent sensors, which can provide a cheaper means of clinical diagnosis of osteoporosis. The MIPs as an artificial antibody can replace antibodies previously used in the ELISA technique and provide a cost-effective method, and quantum dot-incorporated MIPs can help in monitoring bone loss in vivo. As of now most MIPs-based techniques in clinical diagnosis are still under research, and there needs to be a lot of optimization done for the synthesis of MIPs. A proper method for synthesis of MIPs and efficient, large-scale production of MIPs still lies as a challenge for MIPs as syntheses of antibodies have dedicated facilities around and research all around the world, and antibodies have already made their footprint in clinical and practical settings and are biocompatible when compared to MIPs, which are still in the developmental stage when it comes to clinical translation. Biocompatibility of MIPs is still a gap in this field. There is a need for biodegradable MIPs, which only developed recently. MIPs synthesis can sometimes result in the formation of improper polymers with poor polymerization, nonuniform particles, and an uneven size distribution. As polymerization reactions require inorganic solvents, there is a need for hydrophilic polymers, and MIP efficacy should be increased. For now, MIPs target only one target, but in the future, MIPs-based sensors can be designed to target multiple analytes simultaneously.^68^ These MIPs can increase the rate at which people are diagnosed, and these MIPs can easily detect not only bone loss but also other diseases and disorders in the body. MIPs can improve the field of clinical diagnosis by providing cheap and cost-effective alternatives to pre-existing clinical techniques. Hand-held devices like glucometers, portable diagnostic devices, and point-of-care devices can be developed using MIPs in a cost-effective manner and can be developed in the fields of clinical diagnosis and bone loss detection.
